# Haemoglobin A1c: comparing performance of two point of care devices with laboratory analyser

**DOI:** 10.1186/1756-0500-6-540

**Published:** 2013-12-18

**Authors:** Ruziana Mona Wan Mohd Zin, Zati Iwani Ahmad Kamil, Tuan Rosidah Tuan Soh, Mustaffa Embong, Wan Nazaimoon Wan Mohamud

**Affiliations:** 1 Department of Diabetes & Endocrine, Cardiovascular Diabetes and Nutrition Research Centre, Institute for Medical Research, Jalan Pahang, Kuala Lumpur, 50588, Malaysia; 2 Department of Medical, School of Medical Sciences, Universiti Sains Malaysia, Kubang Kerian, Kelantan, 16150, Malaysia; 3 National Diabetes Institute, Jalan SS 3/50, Petaling Jaya, Selangor, 47300, Malaysia

**Keywords:** HbA1c, Point-of-care, Diabetes

## Abstract

**Background:**

Measurement of HbA1c has been widely used for long-term monitoring and management of diabetes control. There is increasing use of point-of-care (POC) devices for measuring HbA1c where quicker results would allow immediate clinical management decisions to be made. Therefore, it is important to evaluate and compare the performance of such devices to the reference laboratory method.

**Findings:**

A total of 274 venous blood was collected from normal healthy adults during the community screening programmes. The performance of POC devices, Afinion and Quo-test were compared to central laboratory HPLC method; Adams A1c HA 8160. Both POC devices showed good correlation to HA 8160 with r = 0.94 (p < 0.001) and r = 0.95 (p < 0.001) for Afinion and Quo-test respectively. The means difference were statistically higher between POC and HA 8160 with 0.23% (95% CI 0.19-0.26, p < 0.001) and 0.29% (95% CI 0.24-0.34, p < 0.001) for Afinion and Quo-test respectively.

**Conclusions:**

Both POC devices could be considered in health clinics for diabetes management but not to be used for the diagnostic purposes.

## Findings

### Introduction

Diabetes mellitus (DM), especially type 2 diabetes has become a major concern globally and imposes debilitating health issues, especially in low and middle income countries. International Diabetes Federation (IDF) currently reported that half of people with diabetes are undiagnosed [1]. The prevalence of type 2 diabetes among Malaysian adults has risen to 22.6% [2] compared to 14.9% in 2006 [3]. Furthermore, onset of diabetes is often left undetected due to no apparent clinical symptoms, and complications may begin 4 to 7 years before clinical diagnosis [4]. However, public screening for diabetes can be challenging as prior arrangement has to be made to ensure subject fasted for 8 to 10 hours.

Measurement of haemoglobin A1c (HbA1c) in blood has been widely used as a routine method for monitoring long term glycaemic status in patients with diabetes mellitus. The HbA1c level provides the clinician the indication of patient’s average glycaemic control over the past two to three months [5]. Large trials such as Diabetes Control and Complication Trial Research [6] and UK Prospective Diabetes Study [7] found that HbA1c levels correlate with the risk of developing diabetes associated micro- and macrovascular complications. More recently, the International Expert Committee has endorsed the use of HbA1c as a diagnostic tool for diabetes [8].

Over the last years, new devices have been developed which allowed rapid HbA1c determination from capillary blood instead of conventional venipuncture [9]. HbA1c determinations using point-of-care (POC) testing required minimal personnel training and can be easily operated by doctors as well as nurses [10]. By prompt availability of results, POC could minimize patient inconvenience by avoiding extra visit to the clinic and immediate treatment could be instituted [11]. Studies have confirmed that immediate feedback of HbA1c results improves glycaemic control in diabetic patients [12-15].

There is a need to ensure POC measurement of HbA1c provides reliable results that are comparable to central laboratory analysis. The aim of this study was therefore to evaluate the performance of two types of POC devices, Afinion and Quo-test HbA1c and compare to Adams A1c HA 8160, a Diabetes Control and Complications Trial (DCCT) aligned cationic-exchange high performance liquid chromatography (HPLC) analyser.

## Materials and methods

### Laboratory method

The central laboratory determined HbA1c using cationic exchange high performance liquid chromatography (HPLC) using Adams A1c HA-8160 (ARKRAY Inc, Kyoto, Japan). All reagents, controls and calibrators used for this method followed National Glycohemoglobin Standardisation Programme (NGSP) guidelines.

### Point-of-care devices method

Both Afinion (Axis-Shield, Oslo, Norway) and Quo-test (Quotient Diagnostics, Surrey, United Kingdom) are based on a boronate affinity binding method, which has been standardised to the International Federation of Clinical Chemistry (IFCC) reference system [16] for HbA1c and aligned to the DCCT standards via the NGSP. The POC systems were designed to operate with ready to use cartridges with results available in 3 minutes. Both devices can accept capillary or venous blood collected by venipuncture into EDTA tubes.

### Blood sample collection

Our subjects were apparently healthy adults who came for community screening for diabetes programme organised by National Diabetes Institute, which was held in Klang Valley. Ethical approval was obtained from The Human Research Ethics Committee, Universiti Sains Malaysia. All subjects gave written informed consent for participation. Venous blood was collected in EDTA tube and was kept at 4°C and analysed within 48 hours. A total of 274 blood samples were collected and analysed for HbA1c using Adams A1c HA 8160, of which 135 samples were simultaneously analysed using Afinion while another batch of 139 samples collected at another occasion, were also analysed for HbA1c using Quo-test. Blood samples were also collected for fasting glucose level and oral glucose tolerance test (OGTT).

### Statistical analysis

Statistical analysis was performed with SPSS software v16.0 (SPSS Inc, Chicago, USA). Paired t-test was used to determine the significant differences between the groups and Pearson linear correlation coefficient was used to determine the method correlation. Bland-Altman plots were generated using Microsoft Excel.

## Results and discussion

**Figure 1:**
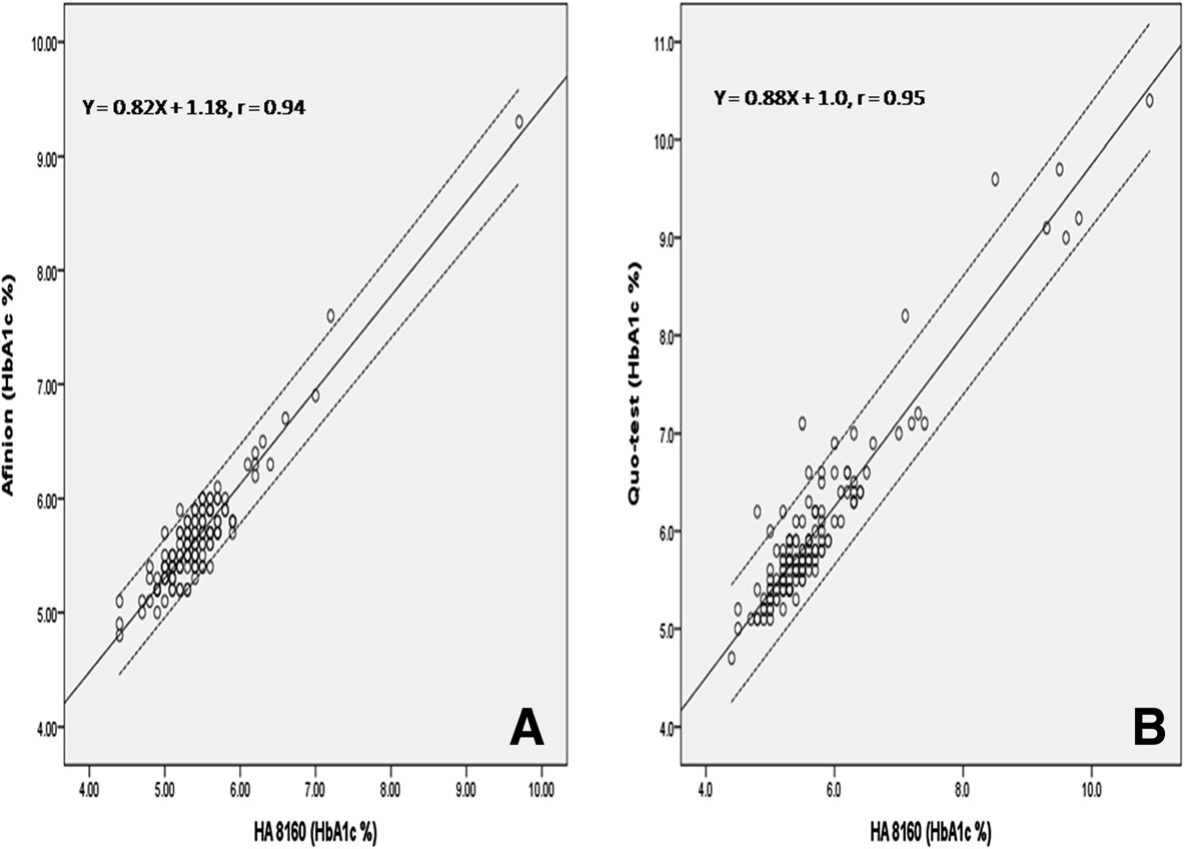
**Method correlation of (A) Afinion and (B) Quo-test compared with HA8160.** The solid line indicates the linear regression whereas the dashed line indicates 95% confidence interval.

**Figure 2:**
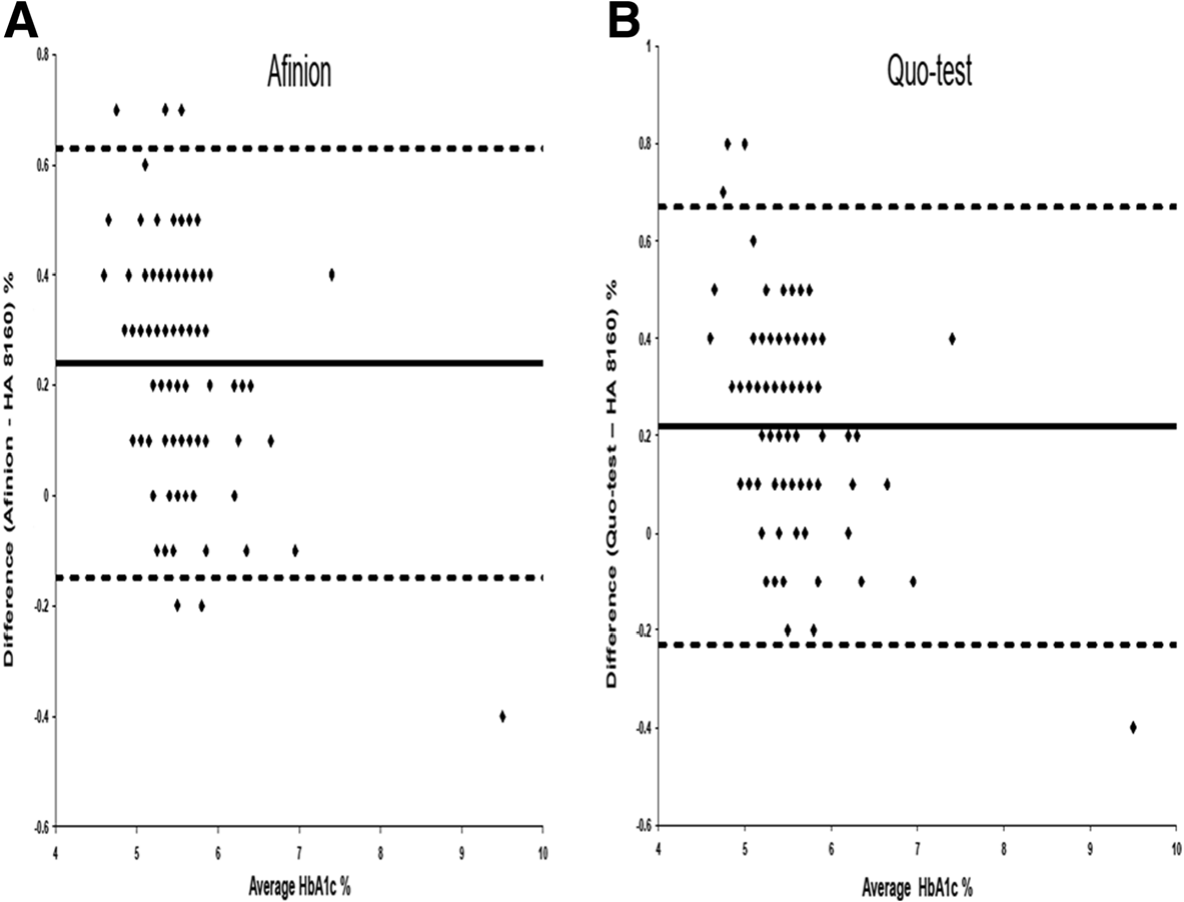
**Bland Altman difference plots of (A) Afinion and (B) Quo-test compared with HA8160.** The solid line indicates the mean difference whereas the dashed lines indicate upper and lower limit of agreement.

Both POC devices showed good correlation to HA 8160 with r = 0.94, p < 0.001 and r = 0.95, p < 0.001 for Afinion (Figure 1A) and Quo-test (Figure 1B) respectively. The means difference were statistically higher between POC and HA 8160 with 0.23% (95% CI 0.19-0.26, p < 0.001) and 0.29% (95% CI 0.24-0.34, p < 0.001) for Afinion and Quo-test respectively (Table 1). Bland-Altman Plot showed 6 samples were out of 2 SD range for Afinion (Figure 2A) and 4 samples were out of 2 SD range for Quo-test (Figure 2B). Based on WHO criteria, 8.1% and 20.9% were found to have diabetes with mean HbA1c of 6.5% and 7.1% for Afinion and Quo-test respectively.

**Table 1:** Comparison of means difference between point-of-care devices and laboratory analyser

	**Mean difference**	**95% confidence interval**	***P***
**Afinion - HA 8160**	0.23	0.19 - 0.26	< 0.001
**Quo-test - HA 8160**	0.29	0.24 - 0.34	< 0.001

Considerable effort has been invested in research and technological development of new POC method that arose from a desire to improve clinical services through a shorter turnaround time for laboratory tests. It has been recognised that POC devices should produce comparable results to laboratory reference method. This study showed that both Afinion and Quo-test devices generated significantly higher HbA1c results compared to HA 8160. In agreement with previously published studies [17,18], this could be attributed by differences in methodology or calibration of devices used. Petersen JR [19] reported that Afinion increasingly underestimated the HbA1c as HPLC HbA1c increased; although management decisions based on HbA1c in the very high range are likely not affected.

It has been recommended that HbA1c assays should have a total intralaboratory imprecision (coefficient of variation, CV) of less than 3% for realistic goal [20] and less than 2% for desirable goal [21]. The CV for Afinion ranged from 0.5% to 2.66% [18,19,22] and from 2.9% to 5.9% for Quo-test [23]. Hence HbA1c results from POC assays are not recommended for diagnosis of diabetes [24].

Studies in Asian populations have shown the optimal diagnostic cut-off point for HbA1c is 6.3% [2,25] instead of 6.5% as recommended by the International Expert Committee. In this study, samples with HbA1c levels higher than 6.3% were from subjects who were diagnosed to have DM based on WHO criteria of fasting plasma glucose ≥ 7.0 mmol/l and/or 2 hour OGTT glucose ≥ 11.1 mmol/l [26]. Hence, HbA1c results from POC assays are suitable for diabetes management.

Although both POC devices showed good correlation in this study, we acknowledge there were few limitations; interference from haemoglobin variants was not evaluated, the study did not include the higher HbA1c levels since samples were from community screening programmes, and diabetes status was determined from a single measurement when ideally diagnosis should be confirmed by repeat testing on a different day.

## Conclusion

In conclusion, both Afinion and Quo-test HbA1c POC devices could be considered in health clinics with minimal laboratory facilities for diabetes management, but not to be used for the diagnostic purposes. POC devices have the advantage of being able to measure HbA1c on site and permit rapid testing using capillary blood samples.

## Competing interest

The authors declare that they have no competing interests.

## Authors’ contribution

RMWMZ participated in data collection, carried out POC analysis and drafted the manuscript. ZIAK participated in data collection and performed statistical analysis. TRTS carried out laboratory analysis. ME and WNWM designed and supervised this study. WNWM revised and approved the final manuscript. All authors read and approved the final manuscript.
